# Association of Osteoarthritis with Perfluorooctanoate and Perfluorooctane Sulfonate in NHANES 2003–2008

**DOI:** 10.1289/ehp.1205673

**Published:** 2013-02-14

**Authors:** Sarah A. Uhl, Tamarra James-Todd, Michelle L. Bell

**Affiliations:** 1 School of Forestry and Environmental Studies, Yale University, New Haven, Connecticut, USA; 2 Brigham and Women’s Hospital and Harvard Medical School, Boston, Massachusetts, USA

**Keywords:** hazardous substances, osteoarthritis, perfluorooctane sulfonate, perfluorooctanoate, public health

## Abstract

Background: Perfluorooctanoate (PFOA) and perfluorooctane sulfonate (PFOS) are persistent, synthetic industrial chemicals. Perfluorinated compounds are linked to health impacts that may be relevant to osteoarthritis, cartilage repair, and inflammatory responses.

Objectives: We investigated whether PFOA and PFOS exposures are associated with prevalence of osteoarthritis, and whether associations differ between men and women.

Methods: We used multiple logistic regression to estimate associations between serum PFOA and PFOS concentrations and self-reported diagnosis of osteoarthritis in persons 20–84 years of age who participated in NHANES during 2003–2008. We adjusted for potential confounders including age, income, and race/ethnicity. Effects by sex were estimated using stratified models and interaction terms.

Results: Those in the highest exposure quartile had higher odds of osteoarthritis compared with those in the lowest quartile [odds ratio (OR) for PFOA = 1.55; 95% CI: 0.99, 2.43; OR for PFOS = 1.77; 95% CI: 1.05, 2.96]. When stratifying by sex, we found positive associations for women, but not men. Women in the highest quartiles of PFOA and PFOS exposure had higher odds of osteoarthritis compared with those in the lowest quartiles (OR for PFOA = 1.98; 95% CI: 1.24, 3.19 and OR for PFOS = 1.73; 95% CI: 0.97, 3.10).

Conclusions: Higher concentrations of serum PFOA were associated with osteoarthritis in women, but not men. PFOS was also associated with osteoarthritis in women only, though effect estimates for women were not significant. More research is needed to clarify potential differences in susceptibility between women and men with regard to possible effects of these and other endocrine-disrupting chemicals.

Perfluoroalkyl acids (PFAAs) are a family of anthropogenic, fluorinated chains of 4–14 carbon atoms (Lau et al. 2007). The unusual oil- and water-repelling characteristics of these molecules led to their use in > 200 industrial processes and consumer applications, including emulsifiers and surfactants; protective coatings for textiles, wood, leather, and metal products; nonstick cookware; grease-proof coatings for paper-based food storage containers; fire-retardant foams; and, personal care products (Lau et al. 2007). Because of their wide range of uses and persistent chemical properties, PFAAs have become ubiquitous contaminants of humans and wildlife (Kuklenyik et al. 2005). Evidence of widespread human contamination with PFAAs was first published about 35 years ago (Guy et al. 1976). More recently, concentrations of specific PFAAs in various environmental media, birds, fish, and humans, have been summarized by Lau et al. (2007). This review of the literature showed that PFAAs have been found in human serum worldwide, and can be measured in wildlife and in fresh and salt water even in remote areas (Lau et al. 2007). These chemicals bioaccumulate, and laboratory data suggest that PFAAs may act as endocrine-disrupting chemicals (Jensen and Leffers 2008). Despite the U.S. Environmental Protection Agency’s (EPA) safety reviews and agreements with some major manufacturers to voluntarily phase-out these chemicals in some locations, use of PFAAs continues, and exposure to many perfluorinated compounds, including PFOA (perfluorooctanoate, or perfluorooctanoic acid) and PFOS (perfluorooctane sulfonate), remains widespread (Calafat et al. 2007).

Osteoarthritis, the most common form of arthritis, affects approximately 27 million adults in the United States (Lawrence et al. 2008) and disproportionately affects women, older individuals, and certain racial/ethnic groups. The disease is characterized by degeneration of tissues in the joints, which leads to chronic pain and joint stiffness. Individuals with osteoarthritis are more likely to report experiencing disability than those without the disease (Botha-Scheepers et al. 2006). The increasing prevalence of osteoarthritis in the United States is likely attributable, at least partly, to the aging population and concurrent increases in overweight and obesity (Bitton 2009). Although the causes of osteoarthritis are not fully understood, inflammation, abnormal calcium homeostasis, and oxidative stress are thought to be involved. In animal and *in vitro* models, PFOA and PFOS have been linked to inflammation (DeWitt et al. 2009; Qazi et al. 2009; Singh et al. 2012), oxidative stress (Eriksen et al. 2010; Qian et al. 2010), and disturbance of calcium homeostasis (Kleszczyński and Składanowski 2011; Liu et al. 2011). In particular, PFOA is hypothesized to increase inflammation through its ability to induce proinflammatory cytokines (Singh et al. 2012). Additionally, by binding to peroxisome proliferator-activated receptor (PPAR)–γ and PPAR-α, PFOA and PFOS could trigger changes in bone metabolism, which could relate to the onset and progression of osteoarthritis symptoms (Innes et al. 2011). A previous study examined the relationships between PFOA and PFOS exposure and osteoarthritis in participants living or working in Ohio and West Virginia communities with PFOA-contaminated drinking water (Innes et al. 2011). In these communities, they found a statistically significant 30% increased odds of self-reported physician-diagnosed osteoarthritis when comparing participants in the highest quartile of PFOA exposure with those in the lowest quartile, whereas they found a negative association for PFOS.

Because the previous study focused on individuals living in highly PFOA-exposed communities (Innes et al. 2011), we aimed to determine whether PFOA and PFOS exposures are associated with increased osteoarthritis prevalence in a population with more common exposure levels for PFOS. Our study participants are a representative sample of individuals from the U.S. population who participated in the National Health and Nutrition Examination Survey (NHANES) from 2003 through 2008. We hypothesized that levels of PFOA and PFOS exposure would be associated with the prevalence of osteoarthritis and that associations would differ by sex because of hormonal differences.

## Methods

NHANES is conducted by the National Center for Health Statistics [Centers for Disease Control and Prevention (CDC), Atlanta, GA], which selects approximately 5,000 representative study participants annually from the civilian, noninstitutionalized U.S. population. NHANES represents the most comprehensive attempt to understand human exposures to chemicals of concern (National Research Council 2006), and has been the sole data source for many cross-sectional studies of associations between chemical exposures and chronic disease. Participants are selected through a multistage probability sampling design. Each of the study participants undergoes a physical examination by a health professional, which includes measurement of height and weight, and completes a series of surveys to ascertain demographic, health, and nutrition information. Various biological samples are also collected for analysis from a random subset of study participants each year. Since 1999, NHANES has operated as a continuous annual survey with data released in 2-year cycles. Further details on study design are available from the CDC (2011a). NHANES was reviewed by the National Center for Health Statistics Ethics Review Board, and documented consent was obtained from participants. The variables used in our analysis are all publicly available through the CDC.

*Exposure.* NHANES has annually assessed perfluorinated compounds since 2003 among a subsample of participants. Perfluorinated compound exposures are estimated by measuring the concentrations of 18 perfluorinated chemicals in serum samples collected from a random sample of one-third of the study participants ≥ 12 years of age (Kuklenyik et al. 2005). In summary, the CDC uses a solid-phase extraction method coupled to high-performance liquid chromatography–tandem mass spectrometry. The limits of detection for PFOA and PFOS are 0.1 and 0.2 μg/g, respectively. At the time of our analyses, laboratory data were available through the 2007–2008 NHANES cycle; we made use of information from 2003–2008 to increase the sample size. We restricted our analyses to persons 20–84 years of age, the group for which we had osteoarthritis status information and precise age information (in NHANES, the ages of all individuals ≥ 85 years of age are coded as 85 years to ensure anonymity). For categorical models of exposure to PFOA or PFOS, we assigned participants to four exposure categories based on distributions in the study population as a whole. The cut points for PFOA were as follows: quartile 1 (≤ 2.95 ng/mL), quartile 2 (> 2.95–4.22 ng/mL), quartile 3 (> 4.22–5.89 ng/mL), and quartile 4 (> 5.89 ng/mL). The cut points for PFOS were as follows: quartile 1 (≤ 8.56 ng/mL), quartile 2 (> 8.56–13.59 ng/mL), quartile 3 (> 13.59–20.97 ng/mL), and quartile 4 (> 20.97 ng/mL).

*Outcome.* Information on the outcome of interest—osteoarthritis status—was collected by questionnaire via self-report. A previous study documented 81% agreement between a self-report of “definite” osteoarthritis and clinical confirmation (March et al. 1998), which suggests that osteoarthritis is likely to have been accurately reported in most cases. All NHANES participants ≥ 20 years of age were asked “Has a doctor or other health professional ever told you that you had arthritis?” Individuals who responded affirmatively were asked a follow-up question: “Which type of arthritis was it?” Possible answers to the latter question included rheumatoid arthritis, osteoarthritis, other type of arthritis, unknown type, and decline to answer the question. Individuals who indicated that a doctor had provided a diagnosis of arthritis, but who declined to answer the question about the type of arthritis or indicated that they did not know which type they had were classified as missing, and were excluded from the analyses. Those who indicated that they had rheumatoid arthritis or a form of arthritis other than rheumatoid or osteoarthritis were considered not to have osteoarthritis.

*Covariates.* Information on potential confounders was obtained from publicly available NHANES data. Potential confounders were selected based on prior reports of associations with PFOA and PFOS exposure levels (Calafat et al. 2007; Nelson et al. 2010) and osteoarthritis (Anderson and Felson 1988; CDC 2011c). We assessed potential confounders as continuous variables unless otherwise noted, including age; sex (male vs. female); poverty status (a ratio of annual family income divided by the federal poverty threshold, calculated by the National Center for Health Statistics); self-reported race/ethnicity (Mexican American, non-Hispanic white, non-Hispanic black, or other, including other Hispanic and multiracial); daily fat and caloric intake (based on responses during the first of two 24-hr dietary recall surveys); body mass index [BMI; weight (kilograms)/height (meters) squared]; self-reported history of bone fractures of the hip, wrist, or spine (yes/no); self-reported participation in moderate or vigorous sports, fitness, or recreational physical activities (yes/no); self-reported smoking status (current, former, never); and for women, self-reported parity (0, 1, or ≥ 2 children). Interpretation of results should consider that further research is needed to disentangle the relationships among some of these covariates, exposure, and health outcome. For example, those with arthritis may engage in less physical activity.

*Statistical analysis*. We used multivariable logistic regression to estimate associations between PFOA and PFOS and odds of osteoarthritis (yes/no). All analyses were conducted separately for PFOA and PFOS. First, we confirmed linear associations between exposure to PFOA or PFOS and odds of osteoarthritis using a test for linear trend. Then we developed models in which the exposures of interest, which were highly right-skewed, were treated as natural logarithm-transformed continuous variables. We developed separate models in which the exposures of interest were treated categorically. We first performed logistic regression with PFOA or PFOS and osteoarthritis without adjustment by any covariates to obtain crude estimates. We then adjusted for sociodemographic factors including age, poverty:income ratio, race/ethnicity, and sex. After eliminating highly correlated dietary and exercise variables, we performed backward model selection using likelihood ratio tests to build fully adjusted models including potential confounders that were statistically significant predictors of the outcome (*p* < 0.05).

We present results for the association between PFOA or PFOS and osteoarthritis based on three models: a crude (unadjusted) model; a model adjusted for sociodemographic factors (age, race/ethnicity, and poverty:income ratio); and a fully adjusted model with adjustment for age, race/ethnicity, and poverty:income ratio, as well as variables selected to be associated with osteoarthritis based on the backward model selection.

We used multiplicative interaction terms and stratified models to assess potential effect modification by sex, age (29–49 years or 50–84 years), and obesity status (BMI ≥ 30 or < 30). All models accounted for the complex, multistage sampling design of NHANES as recommended by the CDC (2011b). Stratum, cluster, and subsample weights were included in all logistic regression models using SAS statistical software survey procedures used in previous analyses of NHANES data (e.g., Meeker and Ferguson 2011; You et al. 2011). All analyses were performed using SAS statistical software version 9.2 (SAS Institute Inc., Cary, NC). Estimates were considered statistically significant based on two-tailed *p*-values < 0.05.

## Results

Of 15,562 individuals 20–84 years of age who participated in NHANES during 2003–2008, PFOA and PFOS exposure information was available for 4,562 individuals, and 4,102 of these individuals also had osteoarthritis status information. Participants with missing information for one or more model covariates (income, BMI, smoking, or history of bone fractures) were excluded. Approximately 6% (*n* = 243) of these 4,102 subjects had missing income information, and were excluded from our analyses. BMI information was missing for about 1.3% of the remaining subjects (*n* = 55). Smoking information was missing for < 1% of subjects (*n* = 2, both of whom had already been excluded due to other missing information). Information on history of bone fractures was missing for one individual who had been excluded due to missing income information.

Our study population included similar numbers of males and females, and had a relatively even age distribution (Table 1), and characteristics were similar to the overall NHANES sample of 15,562 individuals who participated during the study time period (data not shown). Compared with females, males had higher exposures to both PFOA (33.4% higher, *p* < 0.001) and PFOS (38.1% higher, *p* < 0.001). Mean serum PFOA and PFOS concentrations also increased with age (*p* < 0.001), except for a small decline in PFOA in the oldest age group (70–84 years) compared with the next youngest group (Table 1). Exposures also differed by self-reported race/ethnicity for both PFOA and PFOS (*p* < 0.001), with the highest mean PFOA and PFOS concentrations in non-Hispanic whites and non-Hispanic blacks, respectively, and the lowest mean concentrations of both exposures in Mexican Americans. PFOA and PFOS exposures increased with socioeconomic status as indicated by the poverty/income ratio (PFOA: *p* = 0.012; PFOS: *p* = 0.202). Exposure levels generally also increased with BMI for both exposures, though average concentrations were lower in obese participants than in overweight participants. Differences in exposure by smoking status were small, with levels for current smokers 9.0% higher and 5.0% lower than for never-smokers for PFOA and PFOS, respectively.

**Table 1:** Characteristics of study population.

Characteristic	n (% within group)	Osteoarthritis cases [n (%)]	PFOA, weighted mean (ng/mL)a	PFOS, weighted mean (ng/mL)a
Overall	4,102 (100)	365 (100)	4.83	21.23
Sex				
Female	2,068 (50.4)	238 (65.2)	4.22	18.17
Male	2,034 (49.6)	127 (34.7)	5.63	25.10
Age (years)
20–29	825 (20.1)	4 (1.1)	4.69	17.46
30–39	728 (17.8	14 (3.8)	4.73	18.68
40–49	687 (16.8)	31 (8.5)	4.85	20.96
50–59	578 (14.1)	56 (15.3)	5.13	24.43
60–69	620 (15.1)	105 (28.8)	5.48	26.88
70–84	664 (16.2)	155 (42.5)	4.94	27.32
Race/ethnicity				
Mexican American	816 (19.9)	30 (8.2)	3.71	15.11
Other Hispanic	246 (6.0)	11 (3.0)	4.81	17.95
Non-Hispanic white	2,017 (49.2)	271 (74.2)	5.16	22.12
Non-Hispanic black	861 (21.0)	42 (11.5)	4.53	24.73
Other	162 (4.0)	11 (3.0)	4.42	21.11
Poverty:income ratio				
Below poverty line	712 (17.4)	40 (11.0)	4.04	17.19
100–200% poverty line	1,075 (26.2)	96 (26.3)	4.56	21.08
> 200% poverty line	2,072 (50.5)	205 (56.2)	5.20	22.69
Unknown	243 (5.9)	24 (6.6)	4.92	20.40
BMI				
Underweight (≤ 18.5)	69 (1.7)	1 (0.3)	4.13	19.73
Normal weight (18.5–25)	1,163 (28.4)	70 (19.2)	4.72	20.06
Overweight (25–30)	1,458 (35.5)	125 (34.2)	5.19	22.80
Obese (≥ 30)	1,357 (33.1)	161 (44.1)	4.87	21.92
Unknown	55 (1.3)	8 (2.2)	3.31	19.03
Smoking status				
Never	2,163 (52.7)	170 (46.6)	4.78	21.37
Former	1,041 (25.4)	149 (40.8)	4.93	23.35
Current	896 (21.8)	46 (12.6)	5.21	20.31
Unknown	2 (0.1)	0 (0.0)	5.77	25.62
History of bone fractures				
Yes	467 (11.38)	73 (20.0)	5.31	23.01
No	3,634 (88.59)	292 (80.0)	4.86	21.61
Unknown	1 (0.02)	0 (0.0)	4.24	17.43
Vigorous physical activity				
Yes	1,085 (26.45)	44	5.00	20.75
No	3,017 (73.55)	321	4.75	21.55
Moderate physical activity				
Yes	2,155 (52.54)	175 (47.9)	4.98	22.00
No	1,946 (47.4)	190 (52.1)	4.83	21.12
Unknown	1 (0.02)	0 (0.0)	5.60	40.40
Overall ranges: PFOA 0.07–104.00, PFOS 0.14–435.00. aArithmetic mean.				

Osteoarthritis cases were more likely to be female, older, non-Hispanic white, of higher income, and of higher BMI than controls. Exposure to PFOA and PFOS differed by osteoarthritis status, with cases having higher levels than noncases. The survey-weighted mean PFOA exposures for cases and noncases were 5.39 ng/mL (95% CI: 4.91, 5.87 ng/mL) and 4.87 ng/mL (95% CI: 4.59, 5.15 ng/mL), respectively. For PFOS, the survey-weighted mean exposures for cases and non-cases were 24.57 ng/mL (95% CI: 21.49, 27.65 ng/mL) and 21.32 ng/mL (20.05, 22.59 ng/mL), respectively.

In logistic regression models of all participants (males and females), continuous natural logarithm-transformed PFOA and PFOS exposures were positively associated with osteoarthritis without adjustment (Tables 2 and 3). However, associations were not statistically significant after full adjustment, and the OR for PFOS was attenuated toward the null. Comparing subjects in the highest quartile to the lowest quartile of serum PFOA and PFOS, we found statistically significant higher odds of osteoarthritis in the crude (unadjusted) models (Tables 2 and 3). The crude model for PFOA showed increased odds of osteoarthritis with higher exposure. Those in the fourth quartile of PFOA exposure had 62% higher odds [odds ratio (OR) = 1.62; 95% CI: 1.10, 2.39] of osteoarthritis than those in the first quartile. The unadjusted association for PFOS showed some evidence of a dose–response relationship. Study participants in the third and fourth quartiles of PFOS exposure had 2.00 and 2.16 times higher odds of osteoarthritis than those in the first quartile (95% CI: 1.27, 3.17 and 1.37, 3.39), respectively.

**Table 2:** Weighted associations between PFOA exposure and self-reported osteoarthritis in U.S. adults 20–84 years of age.

Exposure	Females and males (n = 3,809)			Females (n = 1,921)			Males (n = 1,888)		
	Crude OR (95% CI)	Adjusted OR 1a (95% CI)	Adjusted OR 2b (95% CI)	Crude OR (95% CI)	Adjusted OR 1a (95% CI)	Adjusted OR 2b (95% CI)	Crude OR (95% CI)	Adjusted OR 1a (95% CI)	Adjusted OR 2b (95% CI)
Quartile 1	Reference	Reference	Reference	Reference	Reference	Reference	Reference	Reference	Reference
Quartile 2	1.60 (1.03, 2.50)*	1.36 (0.84, 2.21)	1.32 (0.78, 2.23)	2.24 (1.43, 3.51)**	1.45 (0.84, 2.50)	1.44 (0.80, 2.62)	0.93 (0.45, 1.92)	1.07 (0.48, 2.36)	0.97 (0.42, 2.27)
Quartile 3	1.42 (0.93, 2.17)	1.18 (0.73, 1.90)	1.20 (0.72, 2.00)	1.93 (1.19, 3.14)**	1.16 (0.68, 1.96)	1.18 (0.67, 2.08)	1.01 (0.55, 1.85)	1.04 (0.50, 2.16)	0.98 (0.46, 2.08)
Quartile 4	1.62 (1.10, 2.39)*	1.45 (0.97, 2.17)	1.55 (0.99, 2.43)	3.71 (2.45, 5.62)**	1.87 (1.22, 2.87)**	1.98 (1.24, 3.19)**	0.70 (0.38, 1.31)	0.80 (0.40, 1.59)	0.82 (0.40, 1.70)
Continuousc	1.28 (1.05, 1.55)*	1.17 (0.96, 1.42)	1.20 (0.96, 1.49)	2.03 (1.58, 2.61)**	1.37 (1.03, 1.71)*	1.35 (1.02, 1.79)*	0.84 (0.65, 1.08)	0.89 (0.68, 1.18)	0.89 (0.67, 1.19)
Results for each sex were obtained from stratified models. aAdjusted for age (continuous), race/ethnicity (non-Hispanic white, non-Hispanic black, Mexican American, other race/multiethnic), socioeconomic status (poverty:income ratio, continuous). bAdjusted for variables above, and smoking (never, former, current), BMI (continuous), vigorous recreational activity (yes/no), prior hip, wrist, or spine fracture (yes/no). cORs represent the relative odds of osteoarthritis associated with a 1-unit increase in in-transformed PFOA. *p < 0.05. **p < 0.01.									

**Table 3:** Weighted associations between PFOS exposure and self-reported osteoarthritis in U.S. adults 20–84 years of age.

Exposure	Females and males (n = 3,809)			Females (n = 1,921)			Males (n = 1,888)		
	Crude OR (95% CI)	Adjusted OR 1a (95% CI)	Adjusted OR 2b (95% CI)	Crude OR (95% CI)	Adjusted OR 1a (95% CI)	Adjusted OR 2b (95% CI)	Crude OR (95% CI)	Adjusted OR 1a (95% CI)	Adjusted OR 2b (95% CI)
Quartile 1	Reference	Reference	Reference	Reference	Reference	Reference	Reference	Reference	Reference
Quartile 2	1.14 (0.67, 1.92)	1.02 (0.59, 1.75)	1.04 (0.58, 1.85)	1.11 (0.61, 2.03)	0.89 (0.48, 1.67)	0.88 (0.46, 1.70)	1.69 (0.57, 5.05)	1.43 (0.46, 4.39)	1.32 (0.41, 4.25)
Quartile 3	2.00 (1.27, 3.17)**	1.80 (1.08, 3.00)*	1.99 (1.14, 3.49)*	2.60 (1.46, 4.63)**	1.74 (0.92, 3.27)	1.92 (0.98, 3.75)	2.20 (0.77, 6.30)	1.90 (0.63, 5.76)	1.86 (0.55, 6.25)
Quartile 4	2.16 (1.37, 3.39)**	1.57 (0.97, 2.54)	1.77 (1.05, 2.96)*	3.31 (1.98, 5.54)**	1.54 (0.90, 2.66)	1.73 (0.97, 3.10)	2.52 (0.98, 6.50)	1.61 (0.62, 4.18)	1.56 (0.54, 4.53)
Continuousc	1.37 (1.12, 1.67)**	1.09 (0.90, 1.32)	1.15 (0.94, 1.40)	1.75 (1.37, 2.23)**	1.14 (0.90, 1.46)	1.22 (0.94, 1.58)	1.34 (0.97, 1.83)	0.95 (0.74, 1.23)	0.95 (0.73, 1.23)
Results for each sex were obtained from stratified models. aAdjusted for age (continuous), race/ethnicity (non-Hispanic white, non-Hispanic black, Mexican American, other race/multiethnic), socioeconomic status (poverty:income ratio, continuous). bAdjusted for variables above, and smoking (never, former, current), BMI (continuous), vigorous recreational activity (yes/no), prior hip, wrist, or spine fracture (yes/no). cORs represent the relative odds of osteoarthritis associated with a 1-unit increase in in-transformed PFOA. *p < 0.05. **p < 0.01.									

These results were generally robust to adjustment by covariates in the partially adjusted model (adjusting for age, sex, race/ethnicity, and income) and the fully adjusted model (adjusting for covariates from the partially adjusted model as well as smoking, BMI, physical activity, and history of bone fractures), although some results lost statistical significance. In our partially and fully adjusted models, those in the fourth quartiles of PFOA and PFOS exposure continued to have elevated odds of osteoarthritis compared to those in the first quartiles of exposure (Tables 2 and 3). After full adjustment, those in the highest quartile of PFOA exposure had a nonsignificant 1.55 times higher odds of osteoarthritis compared with those in the lowest quartile (95% CI: 0.99, 2.43). After full adjustment, those in the highest quartile of PFOS exposure had a 1.77 times higher odds of osteoarthritis compared with those in the lowest quartile (95% CI: 1.05, 2.96).

In general, fully adjusted ORs were stronger for obese participants compared with non-obese participants [see Supplemental Material, Table S1 (http://dx.doi.org/10.1289/ehp.1205673)] though differences were not statistically significant.

**Figure 1:**
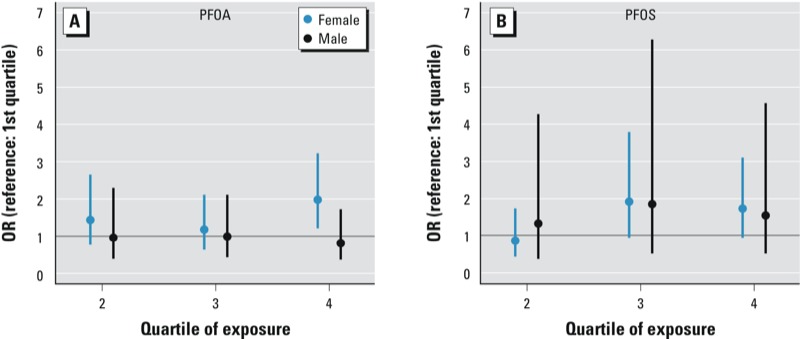
Associations between PFOA (*A*) and PFOS (*B*) exposure quartile (compared with the first quartile; reference) and odds of osteoarthritis, by sex. Points and vertical lines represent effect estimates and 95% CIs from fully adjusted, sex-stratified models, adjusting for age, race/ethnicity, poverty:income ratio, smoking, BMI, vigorous recreational activity, and prior fracture (hip, wrist, or spine).

Stratified models by sex showed slightly stronger associations in women than in men (Figure 1). Fully adjusted ORs comparing the highest with the lowest quartile of PFOA exposure were 1.98 (95% CI: 1.24, 3.19) for women and 0.82 (95% CI: 0.40, 1.70) for men (Table 2). Corresponding ORs for PFOS were 1.73 (95% CI: 0.97, 3.10) for women and 1.56 (95% CI: 0.54, 4.53) for men (Table 3). These results were consistent with models with an interaction term for sex and exposure as a continuous variable, which also indicated stronger associations for females. In these interaction models, the odds of osteoarthritis were 1.51 times higher (*p* = 0.030) for women than men for PFOA, and 1.33 times higher (*p* = 0.097) for PFOS in the fully adjusted model. Interaction models based on quartiles of exposure showed that the odds of osteoarthritis comparing the fourth and first quartiles of exposure were 1.93 times higher (*p* = 0.032) for women than men for PFOA, whereas for PFOS exposure, the odds for women were 1.27 times higher than for men, although not statistically different (*p* = 0.403).

Models stratified by age suggest stronger associations among those 20–49 years of age compared with older participants (50–84 years) for men and women combined, and among women [see Supplemental Material, Table S2 (http://dx.doi.org/10.1289/ehp.1205673)]. The ORs comparing the highest to the lowest quartile of PFOA was 4.95 (95% CI: 1.27, 19.4) in younger women, and 1.33 (95% CI: 0.82, 1.16) in older women, and corresponding ORs for PFOS were 4.99 (95% CI: 1.61, 15.4) in younger women, and 1.30 (95% CI: 0.65, 2.60) in older women. Results were not statistically different between older and younger women, or between men and women, and many strata had small sample sizes. Younger women in the highest quartile of PFOA exposure had 4.95 times higher odds of osteoarthritis compared to those in the lowest quartile of PFOA exposure, after full adjustment (95% CI: 1.27, 19.4). For PFOS, younger women showed a similar increase in the odds of osteoarthritis when comparing those in the highest quartile of exposure with those in the lowest quartile (adjusted OR = 4.99; 95% CI: 1.61, 15.4).

## Discussion

We found statistically significant associations between PFOA and PFOS and osteoarthritis. Positive associations between both chemicals and osteoarthritis were observed in females, but not males, both before and after adjustment for potential confounders. Women with the highest levels of PFOA and PFOS appeared to have 1.98 and 1.73 times higher odds of osteoarthritis, respectively, compared with women in the lowest quartiles of exposure to these chemicals. We estimated stronger associations for younger women (20–49 years) than older women (50–84 years) although these results should be interpreted with caution because of the small numbers of osteoarthritis cases when stratified by sex and age. Innes et al. (2011) reported a stronger relationship between PFOA exposure and osteoarthritis in younger men and women compared with older men and women, for whom a diagnosis would likely have taken place closer to the time of blood sampling. This result and our observation of the strongest associations in younger women suggest the need for follow-up in future studies that could better assess exposure before diagnosis and investigate differences in susceptibility to PFCs and other endocrine-disrupting compounds before and after menopause.

Differences in PFOA exposure levels and study population characteristics complicate the comparison of our results with those of Innes et al. (2011). The PFOA exposure reference categories used by Innes et al. encompass the exposures of most of our study participants and U.S. residents in general. Therefore, their results do not imply the absence of low-dose, potentially nonmonotonic effects of PFOA in the U.S. general population. In contrast to our findings, Innes et al. observed a negative association for PFOS at exposure levels that were quite consistent with those in our study. Although sample size limited our ability to test for effect modification by age and obesity status, which was reported by Innes et al., we did not observe statistically significant differences according to age or obesity in our study population; however, there was some suggestion of stronger associations in younger women than older women, and among obese compared with non-obese participants. Further research is needed to determine whether differences between study populations might be explained by differences in exposure, such that very high PFOA exposures might modify effects of PFOS, for example, or by differences related to race/ethnicity or other characteristics that might modify effects of exposure.

We focused on potential effect modification by sex because of previous animal literature suggesting that effects of PFOA and PFOS on osteoarthritis might be hormonally mediated, as well as evidence that the chemicals might be excreted differently by males and females (Betts 2007). However, other studies did not identify differences in PFOA excretion according to sex (Bartell et al. 2010; Brede et al. 2010).

Although the previous study (Innes et al. 2011) was able to focus on age and BMI differences in a population with very high levels of exposure to PFOA, the present study evaluated the associations between PFOA and PFOS and osteoarthritis among a representative sample of the U.S. population. Our findings suggest that females may be more susceptible than males to effects of perfluorinated compounds.

The biological mechanism(s) by which PFOA and PFOS may cause osteoarthritis are not known, but experimental findings suggest they have the potential to mimic and interact with endogenous hormones, increase the expression of proinflammatory cytokines, and bind with PPARs, which are relevant to biological processes that might influence etiology and progression of osteoarthritis. In particular, PFOA and PFOS can bind to PPAR-α and PPAR-γ (DeWitt et al. 2009; Vanden Heuvel et al. 2006), which are involved with regulation of glucose homeostasis, inflammation, and lipid metabolism and storage (Kersten et al. 2000). Very few studies have reported on sex differences between the associations of PFOA or PFOS and health outcomes in humans. However, a recently published prospective cohort study from Denmark that examined the association between *in utero* PFOA exposure and risk of overweight at 20 years of age found a statistically significant association for females but not for males (Halldorsson et al. 2012).

Limitations of this research include the relatively small sample size, the cross-sectional study design, exposure assessment at a single time point for each participant, self-reported information on the outcome of interest and several covariates, missing data for some study participants, and the lack of information about the date of osteoarthritis onset. The use of exercise as a potential confounder, while included in our fully adjusted model and incorporated in the analysis by Innes et al. (2011), warrants further investigation because arthritis may affect the ability to exercise. Additionally, potential effect modifiers should be examined, such as diabetes and many of the characteristics investigated here (e.g., obesity), with a larger sample size. Due to the relatively long half-lives of PFOA and PFOS (Olsen et al. 2007), the single serum samples likely provide reasonable estimates of long-term exposure. Any exposure misclassification would be random and would be unlikely to differ based on disease status. Still, the single serum measurements could represent exposures following osteoarthritis onset, which could have occurred many years before the survey.

Another potential limitation of our work is that samples were taken at a single time point for each participant, and measured concentrations in these samples may not reflect exposures during etiologically relevant time periods. Evidence from NHANES suggests that PFOS levels decreased in the U.S. population during the study period, whereas levels of PFOA have essentially remained stable (Kato et al. 2011). Thus, if past exposures are more relevant to osteoarthritis than recent exposures, associations based on current PFOS levels may underestimate potential effects.

Although the breadth of variables included in NHANES enabled us to examine and adjust for many potential confounders, residual confounding and possible overadjustment could be sources of bias. Our inclusion of BMI and prior history of bone fractures, which could be on a causal pathway between endocrine-system disruption and development of osteoarthritis, may have introduced bias toward the null.

As new information about the health consequences of PFAAs emerges, patterns of production and usage are changing. Global production of PFOS has dropped considerably compared with 1999 levels following the primary manufacturer’s agreement to end production of the chemical, whereas global production of PFOA has increased during the same period (Lau et al. 2007). Recognizing the growing importance of PFOA, the U.S. EPA launched the PFOA Stewardship Program in 2006: Working with the eight leading manufacturers of PFOA, the U.S. EPA developed a goal of eliminating usage and emissions of the chemical by 2015 (Lau et al. 2007). As these compounds are being used less, at least in some parts of the world, newer PFAAs are entering the global marketplace, dominated by molecules with shorter carbon chains that may be less persistent (Betts 2007). These substitute compounds may present their own health and environmental hazards, and new evidence shows that certain substitutes can undergo chemical transformations in the environment yielding PFOS and PFOA (Betts 2007). Given the ongoing use of PFAAs and the global scale of human and environmental contamination, better understanding of the potential health effects of these chemicals, and of factors that might be used to identify susceptible subpopulations, could help to inform public health policies aimed at reducing exposures or associated health impacts. Future research could investigate the health impacts of newer PFAAs and the degree to which certain groups, such as women, may be particularly susceptible.

## Conclusion

Although production and use of PFOA and PFOS have declined due to safety concerns, human and environmental exposure to these chemicals remains widespread. Better understanding of the health effects of these chemicals and identifying any susceptible subpopulations could help to inform public health policies aimed at reducing exposures or associated health impacts. In this cross-sectional study of a representative sample of the adult U.S. population, PFOA and PFOS exposures were associated with higher prevalence of osteoarthritis, particularly in women. To our knowledge, the present analyses represent the first study of the association between perfluorinated compounds and osteoarthritis in a study population representative of the United Sstates. Future prospective studies are needed to establish temporality and elucidate possible biological mechanisms. Reasons for differences in these associations between men and women, if confirmed, also need further exploration. If replicated, these findings would support reducing exposures to PFOA and PFOS, and perhaps other PFAAs, to reduce the prevalence of osteoarthritis in women, a group that is disproportionately affected by this common chronic disease.

## Supplemental Material

(160 KB) PDF
