# Supplementary material for: Association of Osteoarthritis with Perfluorooctanoate and Perfluorooctane Sulfonate in NHANES 2003–2008
Source: Environ Health Perspect. 2013 Feb 14;121(4):447–52. doi: 10.1289/ehp.1205673 (PMC3620767; doi:10.1289/ehp.1205673)
Supplement: (160 KB) PDF [file ehp.1205673.s001.pdf]

Environmental Health Perspectives doi:10.1289/ehp.1205673

Supplemental Material

Association of Osteoarthritis with Perfluorooctanoate and Perfluorooctane Sulfonate in NHANES 2003–2008

Sarah A. Uhl, Tamarra James-Todd, Michelle L. Bell

Table of Contents

Table S1. Weighted Associations Between PFOA and PFOS Exposure and Self-Reported Osteoarthritis in United States Adults Aged 20-84, Stratified by Obesity Status page 2

Table S2. Weighted Associations Between PFOA and PFOS Exposure and Self-Reported Osteoarthritis in United States Adults, Stratified by Age and Gender page 3

Table S1. Weighted Associations[a] Between PFOA and PFOS Exposure and Self-Reported Osteoarthritis in United States Adults Aged 20-84, Stratified by Obesity Status[b]

Exposure	PFOA		PFOS	
	Obese	*Non-Obese*	*Obese*	*Non-Obese*
Q1	Reference	Reference	Reference	Reference
Q2	1.62 (0.82-3.22)	1.17 (0.57-2.41)	1.08 (0.56-2.06)	0.91 (0.38-2.21)
Q3	1.51 (0.75-3.04)	0.99 (0.53-1.85)	1.59 (0.64-3.94)	2.02* (1.05-3.89)
Q4	2.72** (1.48-5.03)	1.02 (0.62-1.70)	2.60* (1.18-5.73)	1.30 (0.63-2.71)
Log(PFOA/S)	1.62** (1.22-2.15)	0.96 (0.74-1.25)	1.30 (0.96-1.76)	1.06 (0.81-1.38)

[a] Fully adjusted odds ratios (95% confidence intervals). Adjusted for: age (continuous), race/ethnicity (non-Hispanic White, non-Hispanic Black, Mexican American, other race / multi-ethnic), socioeconomic status (poverty-income ratio, continuous), smoking (never, former, current), body mass endex (continuous), vigorous recreational activity (yes/no), and prior hip, wrist, or spine fracture (yes/no). Results are provided comparing exposure in each quartile to the first quartile, and separate results using exposure as a continuous variable.

[b] Obese n = 1,277; non-obese n = 2,532

** $p<0.01$, * $p<0.05$

Table S2. Weighted Associations[a] Between PFOA and PFOS Exposure and Self-Reported Osteoarthritis in United States Adults, Stratified by Age and Gender[b]

**Exposure**	**PFOA**		**PFOS**	
**Females**				
Q2	2.71 (0.93, 7.91)	0.99 (0.54, 1.84)	0.65 (0.19, 2.20)	1.05 (0.46, 2.41)
Q3	1.52 (0.36, 6.39)	0.95 (0.51, 1.75)	1.11 (0.29, 4.30)	1.95 (0.85, 4.48)
Q4	4.95* (1.27, 19.4)	1.33 (0.82, 1.16)	4.99** (1.61, 15.4)	1.30 (0.65, 2.60)
Log(PFOA/S)	2.23 (0.81, 6.12)	1.15 (0.85, 1.57)	2.37** (1.35, 4.16)	1.05 (0.79, 1.40)
**Males**				
Q2	1.36 (0.22, 8.41)	0.84 (0.33, 2.13)	(n/a)	1.28 (0.33, 3.89)
Q3	0.66 (0.10, 4.33)	1.21 (0.58, 2.54)	(n/a)	1.11 (0.31, 3.98)
Q4	0.78 (0.17, 3.72)	0.87 (0.42, 1.78)	(n/a)	1.20 (0.36, 3.94)
Log(PFOA/S)	0.70 (0.34, 1.43)	0.99 (0.75, 1.30)	0.92 (0.51, 1.66)	0.96 (0.69, 1.33)
**All**				
Q2	2.36 (0.84, 6.58)	0.97 (0.57, 1.66)	1.10 (0.29, 4.11)	1.04 (0.53, 2.08)
Q3	1.30 (0.34, 5.04)	1.06 (0.63, 1.76)	2.69 (0.84, 8.61)	1.64 (0.87, 3.09)
Q4	2.18 (0.61, 7.82)	1.19 (0.78, 1.82)	3.76* (1.25, 11.4)	1.29 (0.71, 2.33)
Log(PFOA/S)	1.30 (0.63, 2.66)	1.11 (0.88, 1.39)	1.57 (0.96, 2.56)	1.11 (0.88, 1.39)

[a] Fully adjusted odds ratios (95% confidence intervals). Adjusted for: age (continuous), race/ethnicity (non-Hispanic White, non-Hispanic Black, Mexican American, other race / multi-ethnic), socioeconomic status (poverty-income ratio, continuous), smoking (never, former, current), body mass endex (continuous), vigorous recreational activity (yes/no), and prior hip, wrist, or spine fracture (yes/no). Results are provided comparing exposure in each quartile to the first quartile, and separate results using exposure as a continuous variable.

[b] Numbers of observations: Females: 20–49y n = 1,104, 50–84y n = 817; Males: 20–49y n = 1,010, 50–84y n = 878; All: 20–49y n = 2,114, 50–84y n = 1,695

** $p<0.01$, * $p<0.05$
